# Tear Fluid Calcitonin Gene‐Related Peptide Levels Are Elevated in Glycerol Trinitrate‐Induced Cluster Headache Attacks: Results From an Exploratory Study

**DOI:** 10.1111/ene.70745

**Published:** 2026-09-08

**Authors:** Katharina Kamm, Maria Angelhuber, Adrian Marius Le Prince, Andreas Straube, Ruth Ruscheweyh

**Affiliations:** ^1^ Department of Neurology University Hospital, LMU Munich Munich Germany; ^2^ Department of Gynecology and Obstetrics Klinik Josefinum Augsburg Germany; ^3^ Department of Child and Adolescent Psychiatry, Kbo‐Heckscher‐Klinikum Academic Teaching Hospital of Ludwig‐Maximilians‐Universität Munich Munich Germany

**Keywords:** calcitonin gene‐related peptide, cluster headache, headache, tear fluid

## Abstract

**Background:**

Cluster headache (CH) is a primary headache disorder characterized by severe, unilateral headache attacks accompanied by trigemino‐autonomic symptoms. Calcitonin gene‐related peptide (CGRP) is thought to be released during CH attacks, although the role of CGRP has been questioned. In this exploratory study, we investigated tear fluid CGRP levels during experimentally induced CH attacks using glycerol trinitrate.

**Methods:**

Active episodic and chronic CH patients were screened at study Day 1 and eligible patients received sublingual glycerol trinitrate 0.9 mg at study Day 2. Headache characteristics and tear fluid were sampled before and after intake of GTN. Tear fluid CGRP levels were analyzed using a commercial CGRP ELISA (Cusabio, Wuhan, China).

**Results:**

89 episodic (eCH) and chronic cluster headache (cCH) patients were screened. Of these, 18 CH patients (eCH *n* = 11, 42.6 ± 10.0 years) were included in the analysis. Of 16 CH patients reported an experimentally induced CH attack, tear fluid CGRP levels significantly increased from “baseline” to “headache” (“baseline”: 2.34 ± 3.22 ng/mL, “headache”: 3.70 ± 5.60 ng/mL; *p* = 0.019). Tear fluid CGRP levels increased significantly more in participants with an intense headache attack (NRS ≥ 5: +2.65 ± 3.51 ng/mL) compared to participants with a mild intensity (NRS < 5: +0.07 ± 0.83 ng/mL, *p* = 0.015).

**Conclusion:**

Our study results support the role of CGRP in the pathophysiology of CH. Further studies with a larger study sample and investigation of other neuropeptides are needed to better understand the underlying mechanisms.

## Graphical abstract

Cluster headache (CH) is a severe primary headache disorder and the neuropeptide calcitonin gene‐related peptide (CGRP) has been shown to be elevated ictally and interictally. We investigated tear fluid CGRP levels during experimental cluster headache attacks and showed elevated CGRP levels during these headache attacks.

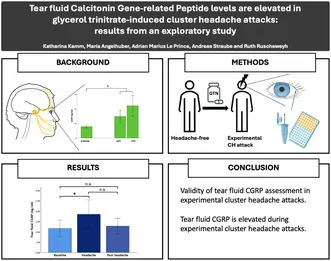

## 1 Introduction

Cluster headache is a primary headache disorder that affects approximately 0.1% of the population [1, 2]. The disorder is characterized by (very) severe, strictly unilateral headache attacks accompanied by trigemino‐autonomic symptoms defined by the International Classification of Headache Disorders, 3rd edition (ICHD‐3) [3].

The headache disorder manifests as episodic cluster headache affecting ca. 85% of patients or as the less frequent chronic cluster headache. Episodic cluster headache (eCH) is defined by headache attacks that occur up to several times a day over a period of weeks to months. Between these episodes, patients are largely symptom‐free. Chronic cluster headache (cCH) is diagnosed if headache attacks occur without remission, meaning without an interruption of at least 3 months/year [3].

The pathophysiology of cluster headache is not fully understood [1, 4]. Based on its clinical presentation, including its chronobiology, unilateral severe headache, and trigemino‐autonomic symptoms, the hypothalamus, the trigemino‐vascular system, and the trigemino‐autonomic reflex appear to play an important role [1, 4].

The headache is caused by activation of the trigemino‐vascular system, which leads to the release of various neuropeptides. The release of calcitonin gene‐related peptide (CGRP) has been most extensively studied; an increase of the neuropeptide in plasma has been demonstrated in spontaneous and experimentally induced cluster headache attacks [5, 6, 7, 8].

The investigation of the disorder is challenging due to its (mainly) episodic occurrence and short attacks with a duration of 15–180 min [4, 5].

In order to investigate the disorder independently of spontaneous headache attacks, the application of glycerol trinitrate (GTN) is a well‐studied model for triggering experimental headache in primary headache disorders [9]. Administration of GTN was mainly investigated in migraine, and only a few studies showed that the administration of GTN leads to cluster headache‐like attacks in active eCH patients (during the episode) and cCH patients, but not in eCH patients in remission [5, 6, 8, 10, 11].

One of the first studies investigated sublingual application of GTN 1 mg in 28 active eCH patients and 15 eCH patients in remission in 1968. All active eCH patients reported a typical CH attack 41 ± 3 min after application of GTN; none of the eCH patients in remission reported an attack after intake of GTN [10].

The neuropeptide CGRP is even less studied in CH patients and study results concerning the detection of CGRP in peripheral blood is inconsistent—both in migraine and cluster headache. The inconsistent study results in peripheral blood were attributed to the dilution of the neuropeptide, distant sampling from the release site and different detection methods [12, 13, 14]. Alternatively, the detection of CGRP in tear fluid has been established. Elevated CGRP levels have been shown in migraine and CH patients [15, 16, 17]. The concentration of CGRP in tear fluid has been shown to be 140× higher compared to plasma CGRP [16].

The aim of the present exploratory study was to investigate the role of tear fluid CGRP in experimentally induced cluster headache attacks in eCH and cCH patients using glycerol trinitrate.

## 2 Methods

### 2.1 Participants

Cluster headache patients were recruited at the outpatient headache center of the LMU university hospital. The study was conducted in accordance with the Declaration of Helsinki and was approved by the LMU ethics committee (19‐818). All participants gave written informed consent. This study is reported in accordance with the STROBE guidelines for observational studies.

Active episodic or chronic cluster headache patients according to the ICHD‐3 criteria aged 18 years or older were included [3]. The use of a stable preventive medication for cluster headache was allowed.

Episodic cluster headache in remission, other headache disorders, wearing contact lenses at study days, pre‐existing neurological, ophthalmological, or severe medical or psychiatric conditions were exclusion criteria.

Further, subjects with known arterial hypertension or a blood pressure of > 140/90 mmHg at one of the study days as well as breast‐feeding or pregnant women were excluded.

Participants were only included if they were headache‐ and abortive medication‐free for at least 5 h prior to study participation and had no contraindications to GTN application like a known allergy or severe hypotension.

### 2.2 Study Procedure

The study was conducted between 10/2020 and 09/2022 at the outpatient headache center of the Department of Neurology of the LMU university hospital.

Participants were investigated at two separate study days. At study Day 1, participants were thoroughly interviewed regarding their cluster headache history, other medical conditions, and regular intake of medication. Blood pressure was measured and tear fluid samples from both eyes were collected as described below (results will be published elsewhere).

At study Day 2, participants were investigated during an experimentally induced cluster headache attack using glycerol trinitrate. Participants presented to our outpatient headache center and “baseline” tear fluid was collected from both eyes. Then, participants received glycerol trinitrate 0.9 mg sublingually and vital signs were recorded every 5 min. Subsequently, patients filled a short headache questionnaire, rating headache intensity on a numerical rating scale (NRS) from 0 to 10 and the presence of cluster headache symptoms, and tear fluid was collected at regular intervals (ca. 30 min, but adapted to the timing of the headache attack and eventual intake of acute medication). Patients were free to use their usual acute medication (AM) at any given moment. If so, an additional sample was collected before the intake of the medication.

On average, 5 ± 2 tear fluid samples were collected per patient. Two hours after spontaneous or drug‐related substantial improvement of headache, patients were discharged. In total, study participants were observed for 2–4 h after glycerol trinitrate application.

Tear fluid was collected from the lateral canthus of both eyes using a plastic capillary (ref. no. 100012, Sanguis, Nümbrecht, Germany) after participants rested supine for 5 min. The plastic capillary was removed after complete filling or a maximum sampling time of 1 min. Much care was taken not to irritate the eye and the procedure was stopped if the eye showed signs of excessive tearing in order to prevent dilution of samples. The filled capillary was immediately immersed in a 1.5 mL tube containing 500 μL of tissue protein extractor solution (TPER; Pierce Rockford, IL), centrifuged for 5 min at 4000 g, and stored at −80°C.

Tear fluid CGRP levels were determined using a commercial CGRP ELISA kit (Cusabio, Wuhan, China; detection range: 1.56–100 pg/mL, minimal detectable dose: 0.39 pg/mL, intra‐assay precision: < 8%, inter‐assay precision: < 10%), following manufacturer's instructions. Duplicate measurements were performed for each sample. Absorbance values were read using a spectrometer (PerkinElmer, USA) and CGRP concentrations were determined from calibration curves using a 4PL fitting. The final CGRP concentration of each sample was calculated as the average of the two measurements. “Baseline” tear fluid CGRP levels did not differ between the right and left eye of CH patients (right eye: 2.30 ± 3.07 ng/mL, left eye: 2.37 ± 3.62 ng/mL; *z* = 0.511, *p* = 0.609), therefore values from both eyes were pooled.

### 2.3 Statistics

The sample size was estimated based on previous study results of CH attack induction rates and own previous study results [5, 6, 8, 10, 11, 17]. Assuming a 5% type 1 error probability and a power of 90%, a sample size of 20 was estimated [18].

Primary endpoints were differences in tear fluid CGRP levels at maximum headache intensity (“headache”) compared to “baseline” and “post‐headache” tear fluid CGRP levels as well as differences of tear fluid CGRP levels according to headache intensity. Differences in tear fluid CGRP levels in participants without headache after GTN application were analyzed as exploratory analysis.

Data is presented as mean ± standard deviation unless stated otherwise. According to the Shapiro–Wilk‐Test, some data were non‐normally distributed. Therefore, non‐parametric tests were used. For comparison of headache intensity and tear fluid CGRP levels at different time points (before GTN application: “baseline”; at maximum headache intensity: “headache”; at the time of lowest headache intensity within 1 h after abortive medication intake or spontaneous improvement: “post headache”), Friedman test was used followed by post hoc Dunn‐Bonferroni tests if appropriate. Effect size and confidence interval were calculated using Kendall's *W* (*W* = χ^2^ *w*/(*N*(*k* − 1))) [19]. For classification, a Kendall's *W* ≥ 0.2, ≥ 0.4 or ≥ 0.6 is considered fair, moderate or substantial [19, 20]. Further, effect sizes *r* (*r* = *z*/𝑁) and confidence intervals for each Dunn‐Bonferroni test was calculated. A *r* ≥ 0.10, ≥ 0.30 or ≥ 0.50 is defined as small, medium or large effect [21]. For comparison of duration until headache onset or intake of acute medication, headache intensity or “baseline” tear fluid CGRP levels between episodic and chronic CH patients, Mann–Whitney‐*U* test was used. Mann–Whitney‐*U* test was also used for comparison of headache intensity and increase in tear fluid CGRP levels between patients with a moderate or mild headache. Effect size and confidence interval was calculated using Cliff's delta (δ = (2 *U*)/(n1n2) − 1) due to small sample size [22]. A Cliff's delta (δ) ≥ 0.15, ≥ 0.33 or ≥ 0.47 is considered to be a small, medium or large effect [19, 22]. Wilcoxon test was used for the comparison of CGRP levels at “baseline” and follow‐up in participants without headache after GTN application. Effect size r was calculated [19, 21]. Due to the limited sample size of three participants, the confidence interval was not calculated. For correlation between GTN‐induced and spontaneous CH attacks, Spearman's rho was used. Statistical analysis was performed using SPSS 29 (IBM Corp., Armonk, NY, USA). Significance was accepted at *p* < 0.05 (two‐tailed).

## 3 Results

89 eCH and cCH patients were screened interictally (study Day 1; eCH *n* = 56, cCH *n* = 33). Of these, 21 CH patients (eCH *n* = 13) participated in study Day 2 and received GTN 0.9 mg sublingually.

Of the 21 participants who received GTN, 85.7% of participants reported headache (eCH: 85.7%, cCH: 85.7%), 66.7% of participants reported trigemino‐autonomic symptoms (eCH: 64.3%, cCH: 71.4%) and 57.1% of participants developed a headache attack fulfilling ICHD‐3 criteria for cluster headache (eCH and cCH: 57.1%). The most frequent accompanying symptoms were nasal congestion (33.3%; eCH: 28.6%, cCH: 42.9%), rhinorrhoea (23.8%; eCH: 21.4%, cCH: 28.6%) and restlessness (23.8%; eCH: 14.3%, cCH: 42.9%).

**FIGURE 1:**
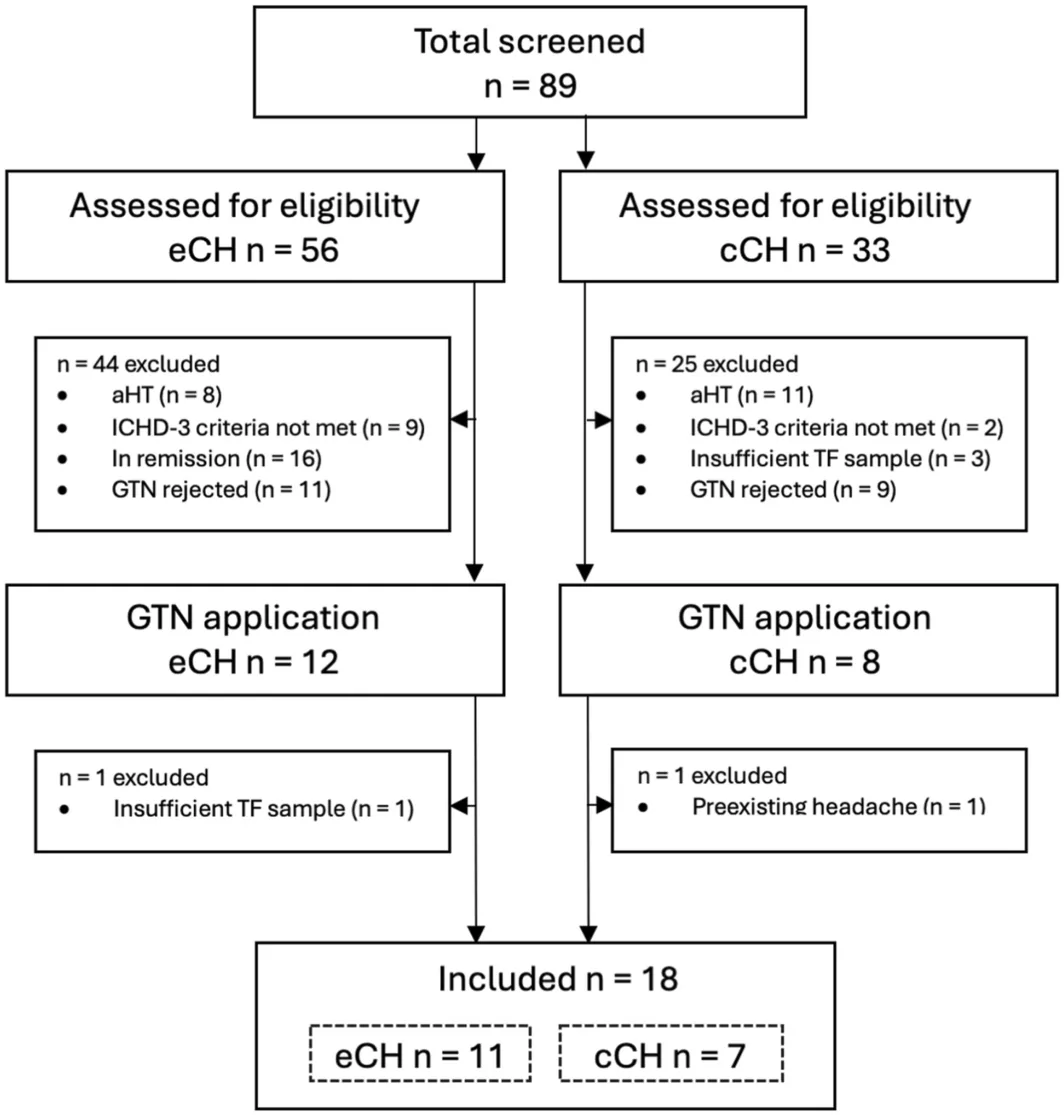
Patient disposition. aHT, arterial hypertension; cCH, chronic cluster headache; eCH, episodic cluster headache; GTN, glycerol trinitrate; ICHD‐3, International Classification of Headache Disorders, 3rd edition; TF, tear fluid.

For further analysis, two participants were excluded from CGRP analysis due to insufficient amount of tear fluid (*n* = 1) or pre‐existing headache at the time of GTN application (*n* = 1, see Figure 1 for patient disposition), leaving 16 CH patients with headache after GTN application and three CH patients without headache after GTN application for tear fluid CGRP analysis (1 patient was investigated with and without headache after GTN application; see Table 1 for demographics).

**TABLE 1:** Demographic and clinical data of cluster headache patients.

	All CH patients	eCH patients	cCH patients	Comparison eCH vs. cCH
N (m %)	18 (77.8%)	11 (90.9%)	7 (57.1%)	p = 0.093^a^
Age (in years)	42.6 ± 10.0	38.8 ± 8.8	48.4 ± 9.4	p = 0.069^b^
Time since first CH attack (in years)	13.5 ± 7.8	13.9 ± 7.4	13.0 ± 8.9	p = 0.813^b^
Episode duration (in weeks)	—	9.2 ± 4.8	—	
Number of episodes since first CH attack, n	—	14.4 ± 8.0	—	
Mean number of headache attacks/day	3.2 ± 3.6	2.1 ± 2.4	4.7 ± 4.5	p = 0.133^b^
Mean headache intensity (NRS 0–10)	8.4 ± 2.1	9.0 ± 1.4	7.6 ± 2.7	p = 0.270^b^
Attack duration (in minutes)	66.4 ± 50.8	81.5 ± 46.8	44.7 ± 51.8	p = 0.070^b^
Abortive medication	18 (100.0%)	11 (100.0%)	7 (100.0%)	p = 1.000^a^
Sumatriptan 6 mg s.c.	9 (50.0%)	5 (45.5%)	4 (57.1%)	
Zolmitriptan 5 mg nasal	9 (50.0%)	7 (63.0%)	2 (28.6%)	
Oxygen	7 (38.9%)	3 (27.3%)	4 (57.1%)	
Prophylactic medication	12 (66.7%)	6 (60.0%)	6 (85.7%)	p = 0.588^a^
Verapamil	9 (75.0%)	4 (66.7%)	5 (71.4%)	
Topiramate	1 (6.3%)	1 (10.0%)	0 (0.0%)	
Medical condition	5 (31.3%)	1 (10.0%)	4 (66.7%)	p = 0.091^a^
Endocrinological condition	2 (12.5%)	0 (0.0%)	2 (33.3%)	
Asthma	1 (6.3%)	0 (0.0%)	1 (16.7%)	
Gastrointestinal condition	1 (6.3%)	1 (10.0%)	0 (0.0%)	
Coagulation condition	1 (6.3%)	0 (0.0%)	1 (16.7%)	
Other regular medication	2 (12.5%)	1 (10.0%)	1 (16.7%)	p = 0.510^a^
Metformin	1 (6.3%)	1 (10.0%)	0 (0.0%)	
Levothyroxine	1 (6.3%)	0 (0.0%)	1 (16.7%)	

Abbreviations: cCH, chronic cluster headache; CH, cluster headache; eCH, episodic cluster headache.

^a^ Fisher's exact test.

^b^ Mann–Whitney‐*U* test.

### 3.1 Characteristics of the Experimentally Induced Cluster Headache Attack

At “baseline”, all 16 included participants were headache‐ and acute medication‐free for at least 5 h (NRS: 0 ± 0). The onset of the headache attack started 39 ± 26 min after intake of sublingual GTN and reached the maximum headache intensity after 46 ± 23 min (range: 15–117 min). Episodic and chronic CH patients did not differ in time until onset of the headache attack (eCH: 38 ± 18 min, cCH: 40 ± 39 min; Mann–Whitney‐*U*‐Test: *U* = 22.500, *p* = 0.428) and time until maximum headache intensity (eCH: 51 ± 27 min, cCH: 39 ± 14 min; Mann–Whitney‐*U*‐Test: *U* = 23.000, *p* = 0.492).

**FIGURE 2:**
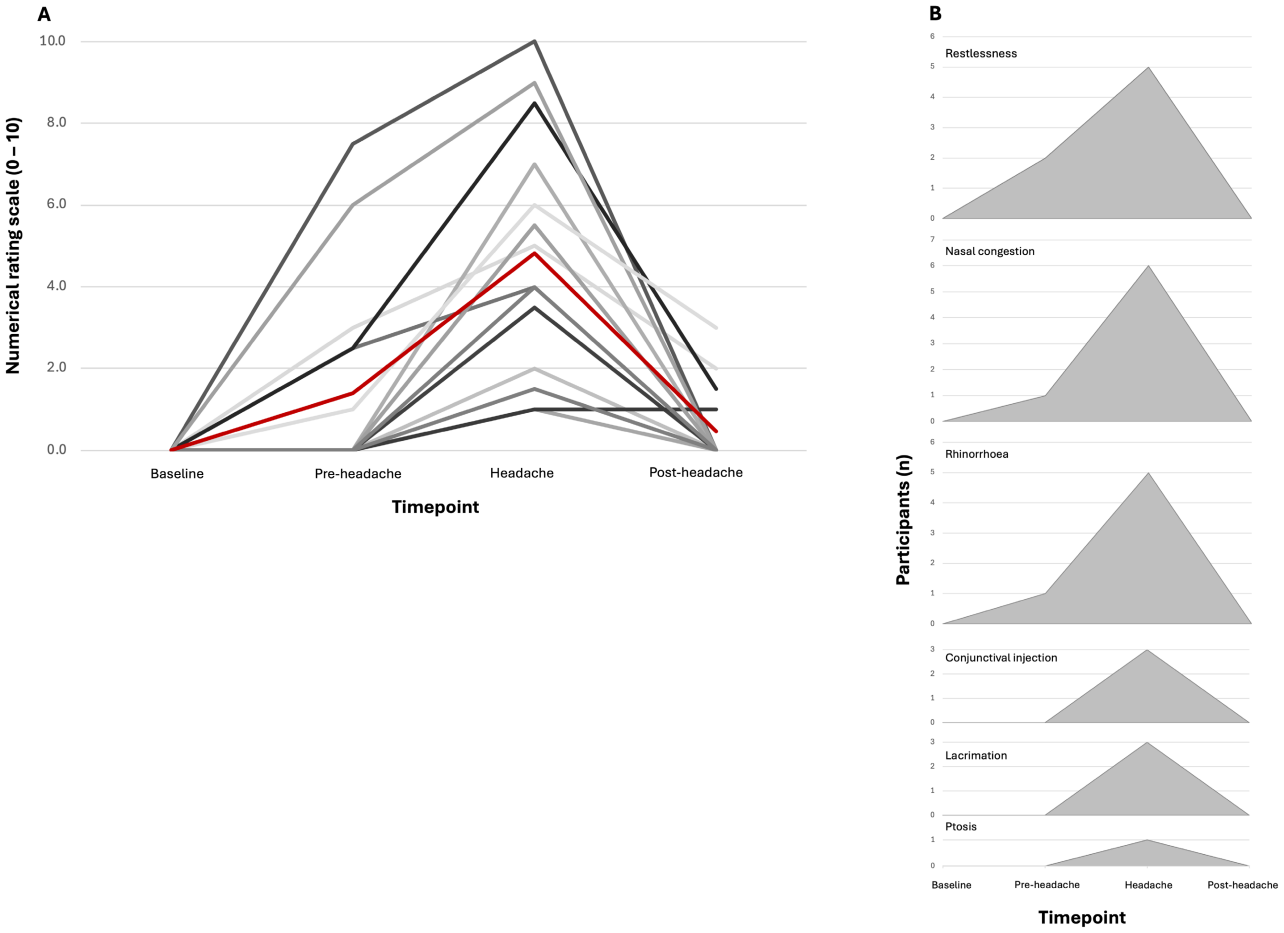
Clinical phenotype of the GTN‐induced cluster headache attack. (A) Individual headache intensities are shown over the course of the CH attack. At “baseline” all participants were headache‐free. At the beginning of the headache attack (“pre‐headache”), the headache intensity rose and peaked at the timepoint “headache”. Spontaneously or after intake of acute medication headache resolved (“post‐headache”). The red line shows the average of the individual headache intensities. (B) Reported accompanying symptoms at the above mentioned time points. NRS, numerical rating scale.

The course of the GTN‐induced headache attack and the accompanying symptoms are shown in Table 2 and Figure 2. The headache intensity during the experimentally induced CH attack was not correlated with the reported usual CH attack intensity (rho = −0.011, *p* = 0.968). 75% of patients reported accompanying symptoms, most often nasal congestion (*n* = 6), restlessness (*n* = 5), or rhinorrhoea (*n* = 5) (see Tables 2 and 3).

**TABLE 2:** GTN‐induced cluster headache attacks in individual patients.

ID	eCH/cCH	Intake of PM	Usual attack intensity (NRS 0–10)	Last CH attack (in h before GTN)	Attack onset (in min after GTN)	Headache intensity (NRS 0–10)	Pain laterality^e^	Accompanying symptoms	AM	Intake of AM (min after GTN)	Intake of AM (min after headache onset)	Headache intensity (NRS 0–10) after resolution
**1** ^a^	eCH	−	10	6	50	8.5	Left‐sided	Lacrimation, nasal congestion, neck pain	Triptan	78	28	1.5
**2** ^a^	eCH	−	10	6	15	5.5	Left‐sided	Restlessness	Triptan	94	79	0
**3**	eCH	+^b^	7	77	45	3.5	Left‐sided	Nasal congestion	Triptan	88	43	0
**4**	eCH	+^d^	6.5	5	15	5	Right‐sided	—	Triptan	92	77	2
**5** ^a^	eCH	+^b^	10	26	41	10	Right‐sided	Conjunctival injection, rhinorrhea, restlessness	Triptan	50	9	0
**6** ^a^	eCH	−	10	120	40	7	Left‐sided	Rhinorrhea	Oxygen	97	57	0
**7**	eCH	+^c^	10	5	45	2	Bilateral	—	—	—	—	0
**8**	eCH	−	7.5	216	70	3.5	Left‐sided	—	—	—	—	0
**9**	eCH	+^b^	10	528	45	4	Left‐sided	Nasal congestion, ptosis	Triptan	97	52	0
**10**	eCH	−	9	18	15	1	Bilateral	Rhinorrhea	—	—	—	0
**11**	cCH	+^b^	8	7	40	1.5	Left‐sided	Rhinorrhea, restlessness, conjunctival injection	—	—	—	0
**12** ^a^	cCH	—	8	12	15	6	Left‐sided	Nasal congestion, restlessness	Triptan	60	45	3
**13**	cCH	+^b^	9	336	117	1	Bilateral	—	—	—	—	1
**14** ^a^	cCH	+^b^	10	6	15	5.5	Left‐sided	Rhinorrhea, lacrimation	Triptan	68	53	0
**15**	cCH	+^b^	10	96	24	4	Left‐sided	Nasal congestion	Oxygen	150	126	0
**16** ^a^	cCH	+^b^	6	144	30	9	Left‐sided	Restlessness, nasal congestion	Triptan	35	5	0

*Note:* The course of GTN‐induced headache attacks in individual participants is shown.

Abbreviations: AM, abortive medication; cCH, chronic cluster headache; eCH, episodic cluster headache; GTN, glycerol trinitrate; PM, preventive medication.

^a^ Headache attack fulfilled ICHD‐3 criteria of cluster headache.

^b^ Participants took verapamil as preventive medication.

^c^ Participant took topiramate as preventive medication.

^d^ Participant took prednisone (40 mg/d) as preventive medication.

^e^ All unilateral attacks were on the side of the patient's usual cluster headache attacks.

**TABLE 3:** Cluster headache‐like headache features of the GTN‐induced headache attack in comparison to the ICHD‐3 criteria in 21 patients.

A.	At least five attacks fulfilling criteria B–D	
B.	Severe or very severe unilateral orbital, supraorbital and/or temporal pain lasting 15–180 min (when untreated)	43.8%
C.	Either or both of the following:	
1. At least one of the following symptoms or signs, ipsilateral to the headache:		75.0%
Conjunctival injection and/or lacrimation		12.5%
Nasal congestion and/or rhinorrhoea		43.8%
Eyelid oedema		0%
Forehead and facial sweating		0%
Miosis and/or ptosis		6.3%
2. A sense of restlessness or agitation		31.3%
D.	Occurring with a frequency between one every other day and 8 per day	
E.	Not better accounted for by another ICHD‐3 diagnosis.	

Abbreviation: ICHD‐3, International Classification of Headache Disorders, 3rd edition.

In seven participants (43.8%; eCH: 40.0%, cCH: 50.0%), the headache attack fulfilled ICHD‐3 criteria for cluster headache. In these patients, the headache intensity was higher compared to patients without fulfilling the ICHD‐3 criteria (NRS: 7.4 ± 1.8 vs. 2.7 ± 1.7; Mann–Whitney‐*U*‐Test: *U* = 63.000, *p* = 0.001).

Eleven patients (68.8%) took acute medication 83 ± 32 min (range: 35–150 min) after intake of GTN; of these, nine patients took triptans (81.8%), while two participants inhaled oxygen. Episodic and chronic CH patients did not differ in time until intake of acute medication (eCH: 86 ± 18 min, cCH: 78 ± 50 min; Mann–Whitney‐*U*‐Test: *U* = 8.000, *p* = 0.476).

### 3.2 Increased Tear Fluid CGRP Levels Over the Course of the Experimentally Induced Cluster Headache Attack

**FIGURE 3:**
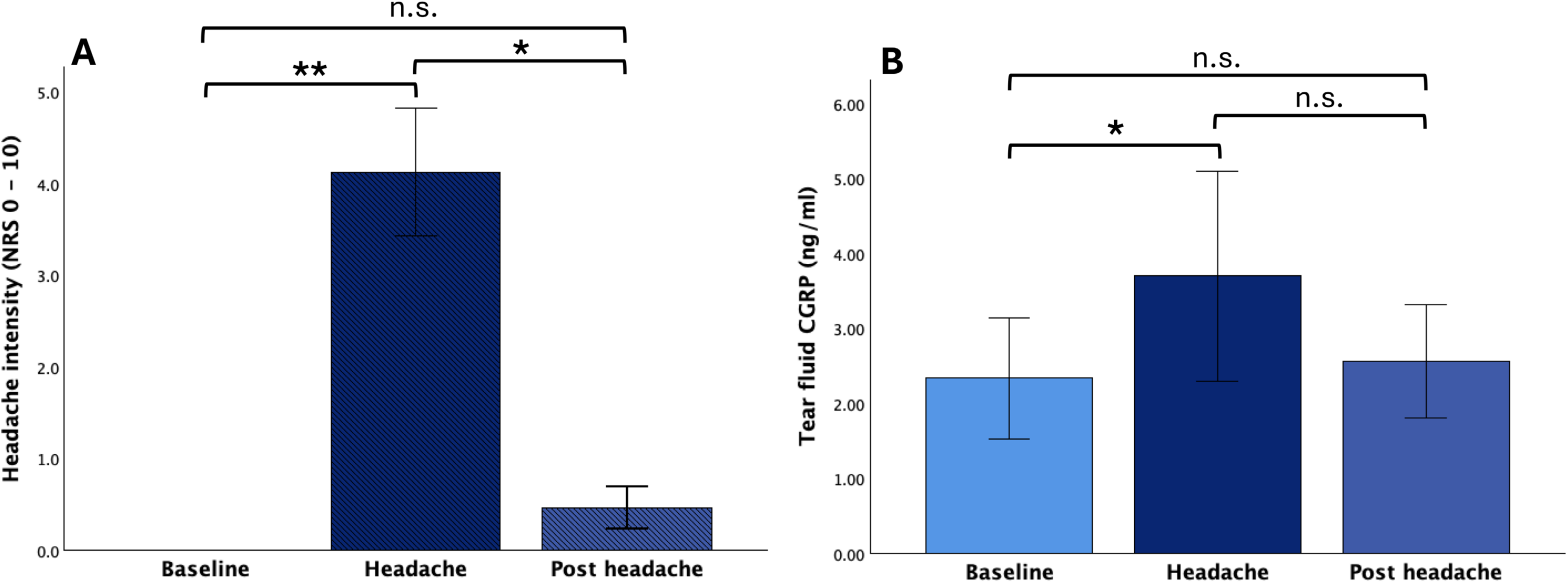
GTN‐induced cluster headache attack. (A) After GTN application, CH patients reported a cluster headache‐like headache attack with a mean headache intensity of 4.8 ± 2.9 on the NRS. After intake of acute medication or spontaneously, headache resolved almost completely (post headache: 0.5 ± 0.9). (B) Tear fluid CGRP was significantly elevated during the headache phase (*p* = 0.024) compared to “baseline” and was normalized after headache resolution. Bars represent standard error. *p < 0.05, **p < 0.001.

For the analysis for tear fluid CGRP, the time points “baseline”, “headache” and “post‐headache” were defined. Headache intensity on the NRS was significantly higher at the “headache” timepoint compared to “baseline” and “post headache” (NRS: “baseline”: 0.0 ± 0.0, “headache”: 4.8 ± 2.9, “post headache”: 0.5 ± 0.9; Friedman test: χ^2^[2] = 27.125, *p* < 0.001, *n* = 16; post hoc Dunn‐Bonferroni test: “baseline” vs. “headache”: *z* = −1.500, *p* < 0.001; “baseline” vs. “post headache”: *z* = −0.281, *p* = 1.000; “headache” vs. “post headache”: *z* = 1.219, *p* = 0.002, see Figure 3A). There was no significant difference in headache intensity at the maximum headache (“headache”) between eCH and cCH patients (NRS: eCH: 4.9 ± 3.0, cCH: 4.5 ± 3.0; Mann–Whitney‐*U*‐Test: *U* = 29.000, *p* = 0.958).

Tear fluid CGRP levels were not significantly different between eCH and cCH patients at “baseline” (eCH: 1.77 ± 1.71 ng/mL, cCH: 3.28 ± 4.91 ng/mL; Mann–Whitney‐*U*‐Test: *U* = 32.000, *p* = 0.875). Therefore, tear fluid CGRP levels of eCH and cCH patients were combined for analysis.

Over the course of the experimentally induced headache attack, tear fluid CGRP levels significantly increased from “baseline” to “headache” (“baseline”: 2.34 ± 3.22 ng/mL, “headache”: 3.70 ± 5.60 ng/mL, “post headache”: 2.57 ± 3.02 ng/mL; Friedman‐Test: χ^2^[2] = 7.875, *p* = 0.019, *n* = 16; post hoc Dunn‐Bonferroni test: “baseline” vs. “headache”: *z* = −0.938, *p* = 0.024, *r* = 0.70, 95% CI [0.31; 0.98]; “baseline” vs. “post headache”: *z* = −0.188, *p* = 1.000, *r* = 0.13, 95% CI [−0.39; 0.59]; “headache” vs. “post headache”: *z* = 0.750, *p* = 0.102, *r* = −0.22, 95% CI [−0.65; 0.31] see Figure 3B). The post hoc test revealed a significant difference between the time points “baseline” and “headache”. The effect size was fair with a Kendall's W = 0.25, 95% CI [0.10; 0.57]. There was no significant difference in tear fluid CGRP increase between eCH and cCH patients (eCH: +0.76 ± 1.49 ng/mL, cCH: +2.37 ± 4.19 ng/mL; Mann–Whitney‐*U*‐Test: *U* = 33.000, *p* = 0.792).

### 3.3 Increase in Tear Fluid CGRP Levels Is Associated With Pain Intensity

**FIGURE 4:**
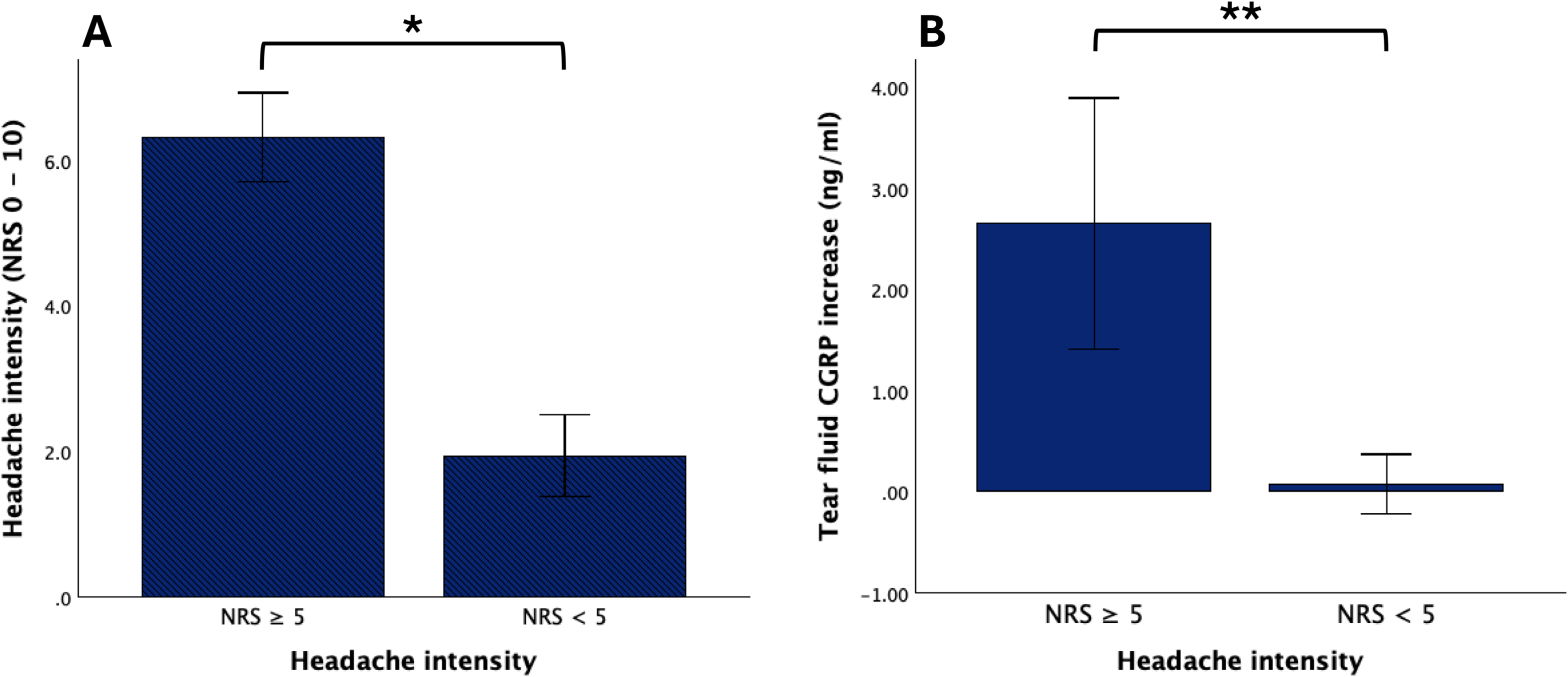
Increase of tear fluid CGRP was higher with more intense headache. (A) Eight participants reported a moderate or intense headache intensity after GTN, while the other eight patients reached a mild headache intensity. (B) In patients with a moderate or intense headache, tear fluid CGRP levels increased significantly more from “baseline” to “headache” compared to patients with a mild headache (*p* = 0.015). Bars represent standard error. *p < 0.05. NRS, numerical rating scale.

Eight participants reported a moderate or intense headache attack (NRS ≥ 5), while the other eight patients experienced a mild headache intensity (NRS < 5). The average headache intensity was significantly different between these patient groups (NRS: NRS ≥ 5: 6.3 ± 1.7, NRS < 5: 1.9 ± 1.6; Mann–Whitney‐*U*‐Test: *U* = 8.500, *p* = 0.010, see Figure 4A). Patients with a moderate or severe headache intensity showed a higher increase in tear fluid CGRP levels compared to those with a mild headache intensity (NRS ≥ 5: +2.65 ± 3.51 ng/mL, NRS < 5: +0.07 ± 0.83 ng/mL; Mann–Whitney‐*U*‐Test: *U* = 9.000, *p* = 0.015, see Figure 4B). Cliff's Delta was δ = −0.72, 95% CI [0.10; 0.94], meaning a large effect.

### 3.4 Unchanged Tear Fluid CGRP Levels in Patients Without Headache After GTN Application

Three participants (eCH *n* = 2, *m* = 3, 44.3 ± 13.5 years) experienced no headache after sublingual GTN application. Baseline tear fluid CGRP levels (1.27 ± 0.44 ng/mL) and tear fluid CGRP levels after 1 h (according to the maximum headache intensity in the headache group) were not significantly different (1.27 ± 0.58 ng/mL; Wilcoxon test: *z* = −0.535, *p* = 0.593, *r* = 0.31).

## 4 Discussion

In this study, we showed significantly elevated tear fluid CGRP levels in experimentally induced cluster headache attacks. The increase in tear fluid CGRP levels was shown in both active episodic and chronic CH patients. After taking acute medication or spontaneous improvement, tear fluid CGRP levels were normalized.

As previously described, sublingual administration of GTN led to a cluster headache attack in active episodic and chronic CH patients approximately 40 min after administration [5]. The time to onset of the headache attack is consistent with previously reported study results [5, 6, 8, 10, 11]. However, we didn't find any differences in time to onset and time to maximum headache between eCH and cCH patients as described before [4].

In our cohort, 57.1% of participants experienced an experimentally induced cluster headache attack fulfilling the ICHD‐3 criteria with no difference between eCH and cCH patients. This percentage is in line with previous study results using sublingual GTN [4]. Trigemino‐autonomic symptoms were reported by 75.0% of patients, nasal congestion and rhinorrhoea being the most often symptoms, partially similar to previous study results [4].

While spontaneous cluster headache attacks were reported to be of maximum or very high intensity in our cohort, GTN‐induced headache attacks were of varying intensity. There was no correlation between both, which might be due to lack of variance in the intensity of spontaneous attacks or due to the nature of experimental attacks. A comparison to former studies investigating GTN‐induced cluster headache attacks is not possible, since headache intensity was mostly not specifically outlined before. One study described headache intensity of spontaneous and GTN‐induced cluster headache attacks, which showed predominantly severe attack intensities. However, intravenous GTN was used in this study, so the clinical phenotype of these experimental attacks should only be compared with caution [11].

The present study demonstrated a significant increase during an experimentally induced headache attack in active cluster headache patients. The increase in CGRP levels is consistent with previous findings, which demonstrated an increase in plasma CGRP in active eCH patients after administration of GTN or spontaneously [5, 6, 7, 8]. New is the evidence that tear fluid CGRP levels are also increased in cCH patients after GTN application, which had not been investigated to date.

Tear fluid CGRP levels were normalized after intake of acute medication. This reduction, however, didn't reach statistical significance. Earlier studies investigating plasma CGRP levels in the external jugular vein found a significant reduction [6, 12]. The missing significance might be due to the limited number of subjects in the present study, or the last tear fluid collection was performed before CGRP levels were fully back to baseline in some patients. In a further study, it would be interesting to expand the time period after intake of acute medication and see whether a further reduction is detectable. Indeed, in a previous study, we found significantly lower tear fluid CGRP levels in CH patients who took recently acute medication compared to interictal patients without acute medication use [17].

Interestingly, three patients without headache after GTN application showed no tear fluid CGRP increase. Although this data has to be interpreted very cautiously due to the small sample size, it might emphasize the association between the activation of the trigemino‐vascular system, CGRP, and headache in CH pathophysiology.

In recent years, the relevance of CGRP in the pathophysiology of cluster headache has been questioned. First, only one placebo‐controlled trial investigating the efficacy of CGRP antibodies for CH prevention (galcanezumab in episodic CH) showed a significant effect, while the trials testing galcanezumab and erenumab in chronic CH and eptinezumab in episodic CH were negative [23, 24, 25, 26]. In addition, although a number of studies showed increased plasma CGRP in cluster headache and during acute CH attacks [5, 6, 8, 27], a recent study showed reduced plasma CGRP levels in CH patients compared to healthy controls [28]. Another study found lower plasma CGRP levels in cCH vs. eCH outside bout patients [29].

In contrast, the present results obtained in tear fluid suggest a role of CGRP in CH attacks. This might be due to tear fluid offering a more direct (less diluted) access to CGRP released from first division trigeminal fibers. Alternatively, lack of control for recent intake of acute medication might have confounded previous results. In our hands, recent intake of acute medication reduced tear fluid CGRP levels to a level indistinguishable from healthy controls [17]. On the other hand, CGRP clearly is not the only neuropeptide involved in CH pathophysiology. Pituitary Adenylate Cyclase‐Activating Polypeptide (PACAP) and Vasoactive Intestinal Peptide (VIP) are thought to play an especially important role because they are released not only from (a subset of) trigeminal fibers but also from parasympathetic fibers involved in the trigemino‐autonomic reflex known to be activated during CH attacks [30, 31, 32, 33]. Elevated PACAP and VIP levels were described during spontaneous CH attacks either compared to remission or healthy controls, although more studies are needed to understand their role in the pathophysiology of CH [7, 34]. The limited effect of CGRP antibodies in cluster headache could further suggest that several neuropeptides act together for a CH attack to occur.

Previous studies and the current findings showed an elevated release of CGRP during an experimentally induced cluster headache attack so it might be assumed that the trigeminal activation also plays a pivotal role in the pathophysiology of cluster headache. Consistent with previous findings, CGRP levels were increased even more if the headache was more intense. Further, this study showed elevated tear fluid CGRP levels in episodic and chronic CH patients during a GTN‐induced CH attack. Interestingly, neither baseline tear fluid CGRP levels nor increase of tear fluid CGRP levels after GTN application were significantly different between eCH and cCH patients, although these results are based on a small sample size (see also the Limitations section). However, these results might indicate a shared pathophysiology of episodic and chronic cluster headache that should be further investigated.

## 5 Limitations

A limitation of this study is the small sample size. With a larger sample size, it would have been possible to better analyse differences between episodic and chronic CH. In future, a larger number of patients should be examined in order to generalize the results. Second, it would have been interesting to perform a cross‐over study design with a randomized GTN or placebo application. Previous studies have shown that GTN can induce an experimentally induced cluster headache attack and that these are associated with elevated plasma CGRP levels in active episodic CH patients compared to episodic CH patients in remission [6, 11]. Further, we could show no tear fluid CGRP change in participants without headache after GTN application, although this evidence has to be discussed cautiously. So, we believe that the tear fluid CGRP elevation is due to the experienced headache attack. However, including a placebo group would have strengthened the study results. Third, for detection of CGRP, the Cusabio ELISA was used which has been questioned regarding its ability to detect CGRP. It was proposed that the Cusabio CGRP ELISA might detect the pro‐peptide rather than ß‐CGRP itself, due to the data sheet as well as negative binding by commercial α‐ or ß‐CGRP [35]. According to the manufacturer's information this ELISA detects ß‐CGRP. To address this issue, we examined tear fluid CGRP levels using the Cusabio CGRP ELISA as well as an additional ‐CGRP ELISA kit (Abbexa, Cambrige, United Kingdom) in a previous study. We could show increased tear fluid CGRP levels during an experimentally induced migraine attack in episodic migraine patients [15]. Taken together, since tear fluid CGRP levels have shown qualitatively similar results using the Cusabio and Abbexa ELISA, we believe that this ELISA can be used to detect CGRP [15, 35]. In a future study, it would be interesting to compare tear fluid CGRP levels in CH patients using different ELISAs to further understand the differences.

## 6 Conclusion

An experimentally induced cluster headache attack with elevation of tear fluid CGRP can be provoked by GTN in episodic and chronic CH patients. This further supports the role of CGRP and trigeminal activation during cluster headache attacks.

### 6.1 Clinical Implications

– Tear fluid sampling is feasible during cluster headache attacks.
– CGRP plays an important role in the acute cluster headache attack of active episodic and chronic cluster headache patients.
– CGRP‐based therapies might be revisited in the treatment of cluster headache patients.

## Author Contributions

**Katharina Kamm:** writing – original draft, investigation, conceptualization, methodology, writing – review and editing, formal analysis, project administration. **Ruth Ruscheweyh:** writing – review and editing, formal analysis, supervision. **Andreas Straube:** conceptualization, writing – review and editing, supervision. **Maria Angelhuber:** investigation, writing – original draft, formal analysis. **Adrian Marius Le Prince:** investigation, writing – original draft, formal analysis.

## Funding

The authors have nothing to report.

## Conflicts of Interest

K.K. has received travel grants and/or honoraria from Lundbeck, TEVA and Novartis. M.A. has no conflicting interests. A.M.L.P. has no conflicting interests. A.S. has received travel grants and/or honoraria from Allergan/AbbVie, Hormosan, Lilly, Lundbeck, Novartis, Sanofi and Teva. R.R. has received travel grants and/or honoraria for lectures or advisory boards, or has consulted for AbbVie, Betapharm, Lundbeck, Novartis, Pfizer and Teva.

## Data Availability

The data that support the findings of this study are available from the corresponding author upon reasonable request.
